# Association Between Admission Calculated Serum Osmolality and New-Onset Atrial Fibrillation in Patients with Non-ST-Segment Elevation Myocardial Infarction Undergoing Percutaneous Coronary Intervention

**DOI:** 10.3390/diagnostics16203365

**Published:** 2026-10-16

**Authors:** Ali Bagci, Hasan Aydın Baş, Yasemin Türker, Fatih Aksoy

**Affiliations:** 1 Department of Cardiology, Faculty of Medicine, Süleyman Demirel University, 32260 Isparta, Türkiye; dr.aksoy@hotmail.com; 2 Department of Cardiology, Isparta City Hospital, Ministry of Health, 32260 Isparta, Türkiye; hasanaydinbas@hotmail.com; 3 Department of Family Medicine, Fatih Family Medicine Center, 32260 Isparta, Türkiye; dryaseminturker@gmail.com

**Keywords:** atrial fibrillation, calculated serum osmolality, NSTEMI, percutaneous coronary intervention, renal function, biomarker

## Abstract

**Background**: New-onset atrial fibrillation (AF) is a common complication of non-ST-segment elevation myocardial infarction (NSTEMI) and is associated with adverse outcomes. We evaluated whether admission calculated serum osmolality is associated with new-onset AF in NSTEMI. **Methods**: This retrospective study included 656 eligible NSTEMI patients undergoing percutaneous coronary intervention (PCI) between March 2021 and April 2022. Only patients in sinus rhythm at admission whose blood sample preceded any AF episode were analyzed. Osmolality was calculated as 2 × sodium + glucose/18 + blood urea nitrogen/2.8. Prespecified logistic models were assessed for discrimination, bootstrap internal validation, calibration, and incremental value. **Results**: New-onset AF developed in 136 patients (20.7%). Osmolality was higher in patients with AF (297.4 ± 8.9 vs. 291.6 ± 15.7 mOsm/kg, *p* < 0.001). In the complete-case model (*n* = 570; 130 events), osmolality was associated with AF (adjusted OR 1.565 per 10 mOsm/kg, 95% CI 1.102–2.223; *p* = 0.012). Adding osmolality increased the AUC from 0.927 to 0.933 (DeLong *p* = 0.081; likelihood-ratio *p* = 0.012; optimism-corrected AUC 0.924). The association was attenuated without renal-function adjustment (*p* = 0.060). **Conclusions**: Admission calculated serum osmolality was associated with new-onset AF in NSTEMI patients undergoing PCI, but its incremental predictive value was modest. External validation is required.

## 1. Introduction

Atrial fibrillation (AF) is the most common sustained cardiac arrhythmia encountered in clinical practice and frequently occurs during the acute phase of acute coronary syndromes (ACS) [1,2]. New-onset AF following non-ST-segment elevation myocardial infarction (NSTEMI) is associated with hemodynamic instability, heart failure, thromboembolic events, prolonged hospitalization, and increased short- and long-term mortality [3]. Despite advances in pharmacological therapy and percutaneous coronary intervention (PCI), AF remains an important marker of clinical vulnerability in NSTEMI patients. Therefore, identifying readily available admission parameters associated with AF development remains clinically relevant.

The mechanisms underlying AF after myocardial infarction are multifactorial. Acute ischemia, atrial pressure overload, autonomic nervous system activation, systemic inflammation, oxidative stress, electrolyte disturbances, and atrial structural remodeling may interact to lower the threshold for atrial electrical instability [4,5,6]. Prior studies have evaluated inflammatory markers, oxidative stress indices, renal function parameters, echocardiographic indices, and clinical risk scores for AF risk stratification; however, many of these variables require additional testing, specialized interpretation, or may not be immediately available in emergency settings [4,5,6,7,8,9].

Calculated serum osmolality is derived from sodium, glucose, and blood urea nitrogen and reflects systemic water–electrolyte balance, metabolic stress, and renal handling of osmotic solutes [10,11,12]. Elevated calculated serum osmolality has been associated with adverse outcomes in acute coronary syndrome, myocardial infarction, heart failure, and critically ill populations [10,11,13,14]. In patients with ACS undergoing PCI, plasma osmolality has previously been linked to worse clinical outcomes, supporting its relevance as an integrated marker of acute physiological stress [11].

Biologically, hyperosmolality may contribute to endothelial dysfunction, neurohormonal activation, sympathetic stimulation, oxidative stress, and inflammatory signaling. Experimental and translational studies suggest that osmotic and inflammatory stress can activate NF-κB, p38 MAPK, and NLRP3-related pathways, thereby promoting cytokine release, fibroblast activation, extracellular matrix deposition, and atrial remodeling [15,16,17,18,19,20,21,22,23]. These mechanisms provide a plausible link between osmolality disturbances and atrial arrhythmogenesis.

Although serum/plasma osmolality has been studied as a prognostic marker in several cardiovascular and critical-care settings, evidence regarding its association with new-onset AF after NSTEMI remains limited. In addition, whether calculated serum osmolality provides information beyond conventional clinical, laboratory, and echocardiographic variables requires careful assessment using temporally appropriate and clinically justified models.

Therefore, the present study aimed to evaluate the association between admission calculated serum osmolality and the development of new-onset AF in NSTEMI patients undergoing PCI. We also sought to assess whether calculated serum osmolality remains associated with new-onset AF after adjustment for clinically relevant covariates and to examine the apparent and internally validated performance of models with and without calculated serum osmolality.

## 2. Materials and Methods

### 2.1. Study Population

This retrospective observational study included eligible patients admitted with non-ST-segment elevation myocardial infarction (NSTEMI) who underwent percutaneous coronary intervention (PCI) at our tertiary cardiovascular center between March 2021 and April 2022. A total of 656 patients were included in the final de-identified analysis cohort. Patients were classified according to the occurrence of new-onset AF during the index hospitalization: patients without AF (*n* = 520) and patients who developed new-onset AF (*n* = 136).

NSTEMI was diagnosed according to the 2020 European Society of Cardiology (ESC) Guidelines for the management of acute coronary syndromes in patients presenting without persistent ST-segment elevation, based on clinical presentation, electrocardiographic findings, and elevated cardiac troponin concentrations [24]. New-onset AF was defined according to the 2020 ESC Guidelines for the diagnosis and management of AF as an irregularly irregular rhythm without distinct P waves lasting at least 30 s on continuous electrocardiographic monitoring or a standard 12-lead electrocardiogram during hospitalization in patients without a previous history of AF [25].

To establish temporal precedence, only patients who were in sinus rhythm on the admission ECG and who had no documented AF before admission blood sampling were included in the new-onset AF analysis. Patients presenting with AF on the admission ECG or developing AF before the admission blood sample used for calculated serum osmolality were excluded from this analysis before construction of the final analytic cohort.

All admission blood samples used to calculate serum osmolality were obtained immediately after hospital presentation and before coronary angiography and PCI. New-onset AF was classified only when the first documented AF episode occurred after the admission blood sample. The available dataset contained time from admission to AF onset; time-stamped PCI and echocardiography data were not sufficiently complete for use in the primary admission model.

AF ascertainment was based on continuous in-hospital ECG telemetry during the coronary care unit stay and standard 12-lead ECG recordings obtained at admission, after PCI when clinically indicated, and whenever symptoms or telemetry findings suggested arrhythmia. The median time from admission to first documented AF was 16.0 h (IQR 3.0–24.0). Median in-hospital monitoring duration was 2.0 days (IQR 2.0–2.0) in patients without AF and 2.5 days (IQR 1.0–4.0) in patients with new-onset AF. Monitoring intensity was protocol-based during the coronary care unit stay, although total monitoring duration varied according to hospitalization course.

Patients with a documented history of atrial fibrillation or flutter, AF on the admission ECG, AF occurring before admission blood sampling, permanent pacemaker rhythm, severe valvular heart disease, active infection, autoimmune or inflammatory diseases, malignancy, severe hepatic dysfunction, end-stage renal disease requiring dialysis, hematological disorders, or missing data required for calculated serum osmolality or multivariable modeling were excluded from the relevant analysis.

The study was conducted in accordance with the Declaration of Helsinki and approved by the Süleyman Demirel University Faculty of Medicine Clinical Research Ethics Committee (Approval No. 25/365, 17 November 2020). Ethics committee approval was obtained before the start of the study period to allow the systematic recording of clinical, laboratory, electrocardiographic, and echocardiographic data from eligible NSTEMI patients undergoing PCI during routine care; the present analysis was subsequently performed retrospectively using this institutional dataset. Patient consent was waived by the Ethics Committee due to the retrospective observational design, use of anonymized clinical data, and absence of any intervention beyond routine clinical practice.

### 2.2. Clinical and Laboratory Assessment

Baseline demographic characteristics, cardiovascular risk factors, medical history, physical examination findings, laboratory parameters, and echocardiographic measurements were obtained from the institutional electronic medical records.

Venous blood samples were collected immediately after admission, before coronary angiography and PCI, and before the first documented AF episode in patients classified as having new-onset AF. Routine biochemical analyses included serum glucose, creatinine, blood urea nitrogen (BUN), electrolytes, hemoglobin, high-sensitivity C-reactive protein (hs-CRP), uric acid, lipid profile, gamma-glutamyl transferase (GGT), cardiac troponin, and creatine kinase-MB (CK-MB).

Calculated serum osmolality was estimated using the following validated formula [11,12]:

Calculated serum osmolality (mOsm/kg) = (2 × Sodium [mmol/L]) + (Glucose [mg/dL]/18) + (Blood Urea Nitrogen [mg/dL]/2.8)

Heart rate, systolic and diastolic blood pressure, body mass index (BMI), and other clinical characteristics were recorded at admission.

### 2.3. Echocardiographic Evaluation

Comprehensive transthoracic echocardiography was performed within the first 24 h of hospitalization using commercially available ultrasound systems by experienced cardiologists as part of routine clinical care, without knowledge of the study hypothesis. The timing of echocardiography relative to AF onset was reviewed to minimize temporal leakage when echocardiographic variables were evaluated as baseline covariates.

Left ventricular ejection fraction (LVEF) was calculated using the modified biplane Simpson method. Left atrial diameter was measured from the parasternal long-axis view according to the recommendations of the American Society of Echocardiography and the European Association of Cardiovascular Imaging [26].

Because echocardiography was performed within 24 h of hospitalization and could occur after AF onset in some patients, echocardiographic variables were not included in the primary admission model. Instead, an extended model additionally including LVEF and left atrial diameter was evaluated as a sensitivity analysis.

### 2.4. Study Endpoint

The primary endpoint of the study was the occurrence of new-onset atrial fibrillation during the index hospitalization.

### 2.5. Statistical Analysis

Statistical analyses were performed using IBM SPSS Statistics software (Version 23.0, IBM Corp., Armonk, NY, USA) and R software (R Foundation for Statistical Computing, Vienna, Austria; https://www.R-project.org/, accessed on 7 October 2026) for model validation, calibration, and ROC comparison analyses. A two-sided *p* value < 0.05 was considered statistically significant. Model reporting followed principles recommended for multivariable prediction-model studies [27].

Continuous variables were assessed for distribution using visual inspection of histograms, Q-Q plots, and normality tests. Normally distributed variables are presented as mean ± standard deviation, whereas non-normally distributed variables are presented as median (interquartile range). Categorical variables are presented as frequencies and percentages. Between-group comparisons were performed using the independent-samples Student *t*-test or Mann–Whitney U test for continuous variables and the chi-square test or Fisher’s exact test for categorical variables, as appropriate.

The multivariable covariate strategy was clinically prespecified rather than based solely on univariable *p*-value screening. Candidate variables were selected according to biological plausibility, prior literature, and availability at admission, including age, sex, BMI, systolic blood pressure, heart rate, renal function (eGFR), potassium, hs-CRP, uric acid, and calculated serum osmolality. Echocardiographic variables were evaluated in an extended sensitivity model because their timing could not be guaranteed to precede AF onset in all patients. Diabetes mellitus, hypertension, and current smoking were evaluated in an expanded clinical sensitivity model to address conventional AF-related risk factors that differed between groups.

Because sodium, glucose, and BUN are direct components of the calculated serum osmolality equation, these individual components were not entered simultaneously into the same multivariable model containing calculated serum osmolality to avoid mathematical coupling and collinearity. Sensitivity analyses compared alternative model specifications, including models using the individual osmolality components instead of the composite variable, models replacing eGFR with creatinine, a model without renal-function variables, and an expanded clinical model adding diabetes mellitus, hypertension, and current smoking.

Multicollinearity was evaluated using correlation diagnostics and variance inflation factors (VIFs), with VIF values > 5 considered to indicate substantial multicollinearity. VIF values for all retained covariates are reported in the Results. Model discrimination was assessed using the area under the receiver operating characteristic curve (AUC). Because the final model was developed and evaluated in the same cohort, bootstrap internal validation with 1000 resamples was performed to estimate the optimism-corrected AUC.

Calibration was evaluated using a calibration plot, calibration intercept, calibration slope, Brier score, and Hosmer–Lemeshow goodness-of-fit test. Because the apparent calibration intercept and slope of a logistic regression model are 0 and 1 by construction when evaluated in its development data, these values are reported for completeness only, and model optimism was quantified by bootstrap internal validation. To assess the incremental value of calculated serum osmolality, a clinically defined base model without osmolality was compared with the same model after adding calculated serum osmolality. Model improvement was assessed using change in AUC, the DeLong test, the likelihood-ratio test, and calibration metrics.

## 3. Results

### 3.1. Baseline Clinical Characteristics

**Figure 1:**
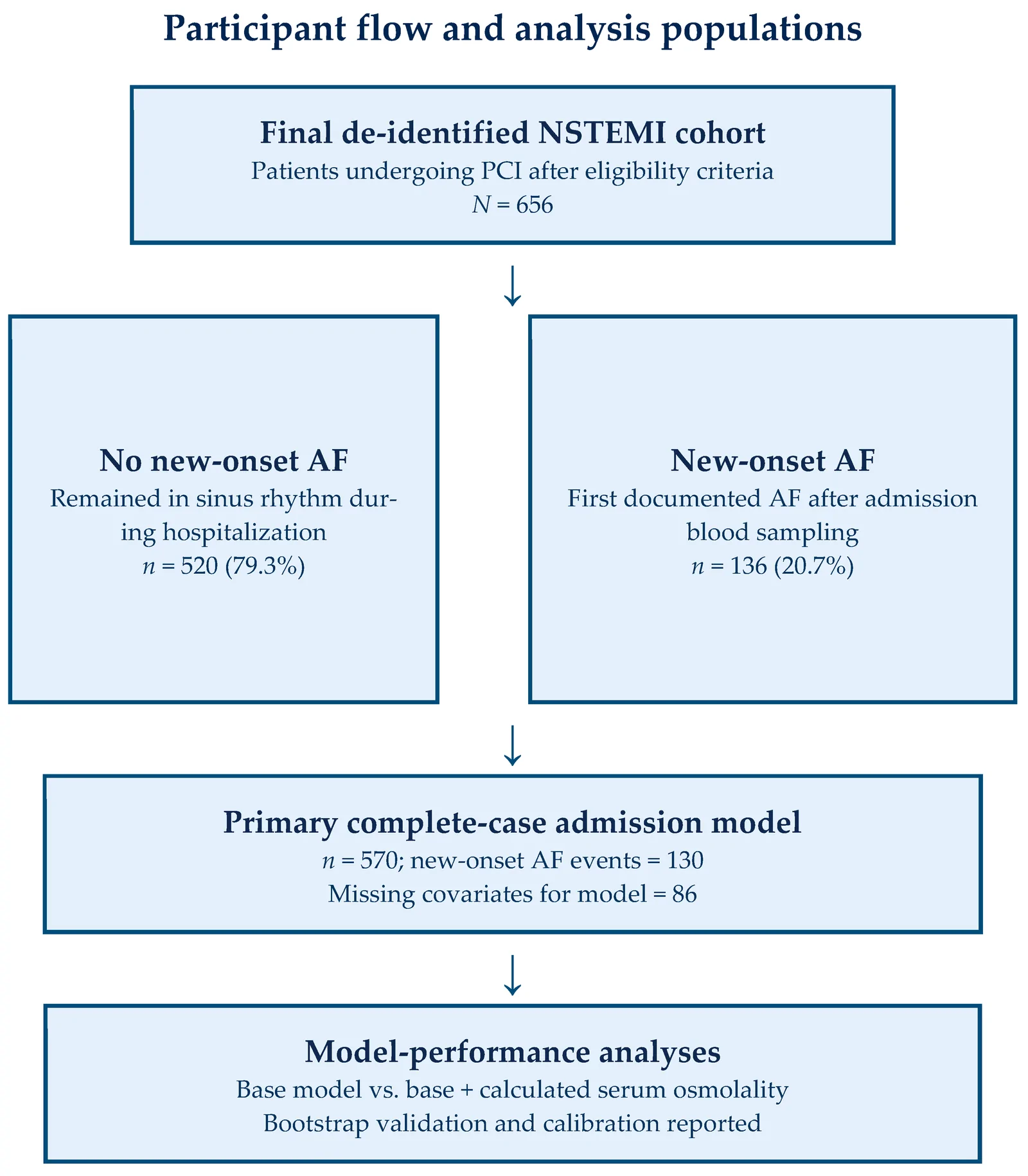
Participant flow diagram and analysis populations. Note: The flow diagram begins with the final de-identified eligible NSTEMI cohort available for analysis. Because the source screening log before de-identification was not fully reconstructable, pre-cohort screening and exclusion counts are not reported.

A total of 656 eligible patients with NSTEMI who underwent PCI were included in the final descriptive analysis (Figure 1). During the index hospitalization, 136 patients (20.7%) developed new-onset AF after admission blood sampling, whereas 520 patients (79.3%) remained in sinus rhythm. Complete-case analysis for the primary admission model included 570 patients with 130 new-onset AF events. The remaining 86 patients (13.1%) were excluded from the multivariable analysis because of missing values in one or more model covariates. Missingness was more frequent among patients without AF than among those with new-onset AF (80/520 [15.4%] vs. 6/136 [4.4%]); consequently, the proportion of AF events was higher in the complete-case population than among excluded patients (22.8% vs. 7.0%).

The admission blood sample preceded the first documented AF episode in all patients classified as having new-onset AF. The median time from admission to first documented AF was 16.0 h (IQR 3.0–24.0). Time-stamped PCI and echocardiography data were not sufficiently complete for use as temporal baseline covariates in the primary model; therefore, echocardiographic variables were analyzed in an extended sensitivity model.

All patients underwent continuous ECG telemetry during the coronary care unit stay. Median monitoring duration was 2.0 days (IQR 2.0–2.0) in patients without AF and 2.5 days (IQR 1.0–4.0) in patients with new-onset AF. Median AF episode duration among AF-positive patients was 10.0 h (IQR 3.0–48.0).

Patients who developed AF were significantly older than those without AF (68.2 ± 11.7 vs. 61.1 ± 12.7 years, *p* < 0.001). The prevalence of diabetes mellitus (35.3% vs. 23.5%, *p* = 0.007), hypertension (72.1% vs. 38.8%, *p* < 0.001), obesity (47.1% vs. 34.2%, *p* = 0.008), and female sex (42.6% vs. 15.4%, *p* < 0.001) was significantly higher in the AF group. Conversely, current smoking was less common among patients who developed AF (44.1% vs. 65.8%, *p* < 0.001).

Regarding hemodynamic measurements, patients with AF had significantly lower systolic blood pressure (109.2 ± 31.0 vs. 124.0 ± 26.9 mmHg, *p* < 0.001) and diastolic blood pressure (64.8 ± 19.3 vs. 74.2 ± 14.6 mmHg, *p* < 0.001). Body mass index was significantly higher in the AF group (29.4 ± 5.2 vs. 26.1 ± 3.6 kg/m^2^, *p* < 0.001) (Table 1).

### 3.2. Laboratory Findings

Patients who developed AF demonstrated greater biochemical evidence of inflammation, metabolic disturbance, and myocardial injury. Because hs-CRP, triglycerides, GGT, CK-MB, and troponin showed skewed distributions, these variables are presented as median (interquartile range) and were compared using the Mann–Whitney U test (Table 1).

Admission calculated serum osmolality was higher in patients who developed AF than in those who remained in sinus rhythm (297.4 ± 8.9 vs. 291.6 ± 15.7 mOsm/kg, *p* < 0.001; Table 1).

### 3.3. Echocardiographic Findings

Patients with AF had significantly larger left atrial diameters compared with patients without AF (42.3 ± 2.7 vs. 39.4 ± 3.4 mm, *p* < 0.001). In addition, left ventricular systolic function was lower in the AF group, as demonstrated by lower LVEF (38.0 ± 10.3% vs. 45.1 ± 9.5%, *p* < 0.001).

### 3.4. Multivariable Associations and Model Performance

In the complete-case admission model, calculated serum osmolality was associated with new-onset AF per 10 mOsm/kg increase (adjusted OR 1.565, 95% CI 1.102–2.223; *p* = 0.012). For clinical interpretability, the association is reported per 10 mOsm/kg increase. Complete univariable and multivariable estimates, *p* values, and VIF values are shown in Table 2.

A clinically prespecified multivariable logistic regression model was constructed to evaluate the association between calculated serum osmolality and new-onset AF. Age was included because it is a major determinant of incident AF and differed substantially between groups. LVEF and left atrial diameter were not included in the primary admission model because echocardiography could not be verified to precede AF onset in all patients; these variables were therefore evaluated in an extended sensitivity model. In this extended model (*n* = 560), calculated serum osmolality remained associated with new-onset AF (adjusted OR 1.543 per 10 mOsm/kg, 95% CI 1.073–2.218; *p* = 0.019), and the model yielded an AUC of 0.948 (Table 3).

**Figure 2:**
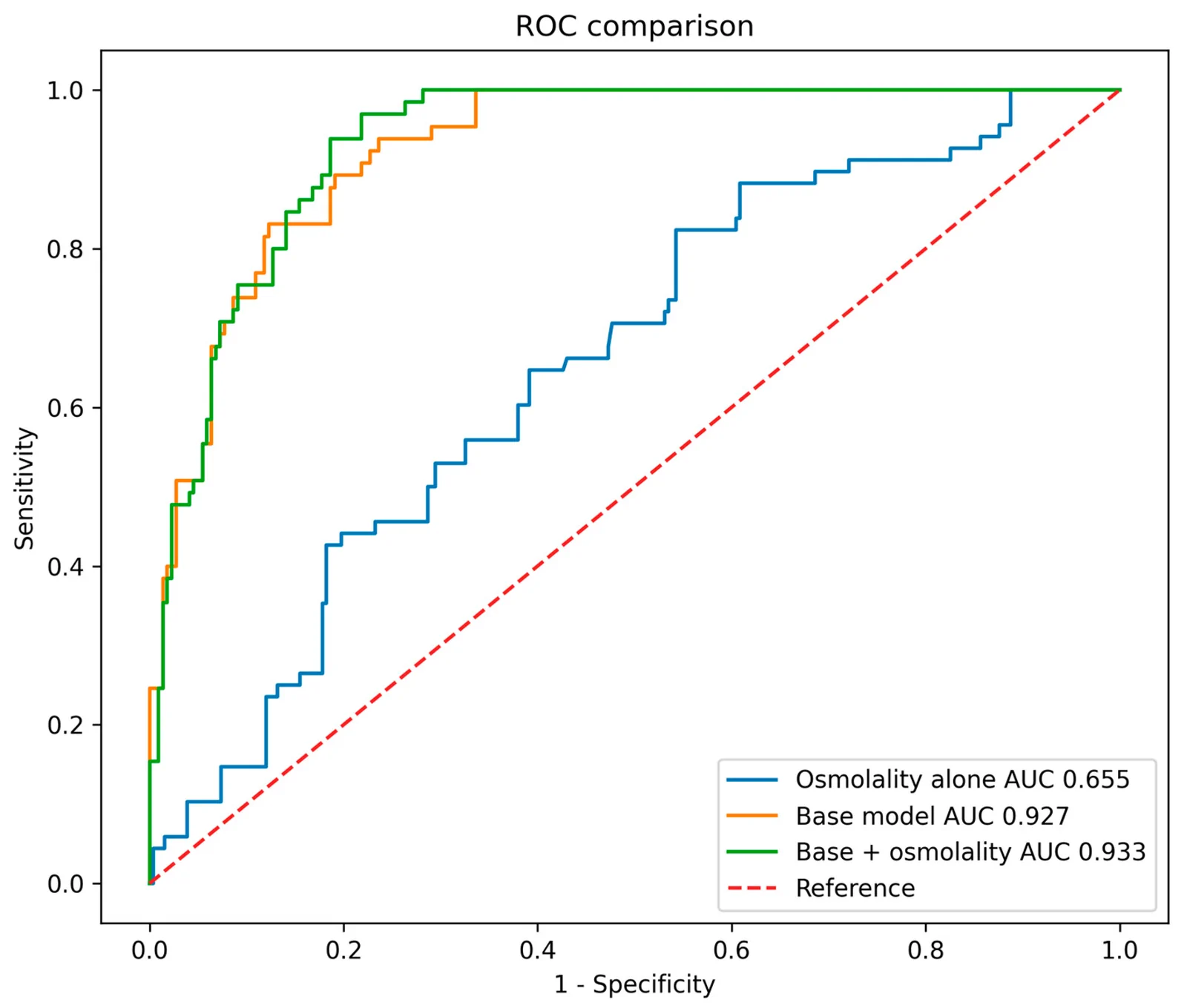
ROC comparison of calculated serum osmolality alone, the base clinical model, and the base clinical model plus calculated serum osmolality.

**Figure 3:**
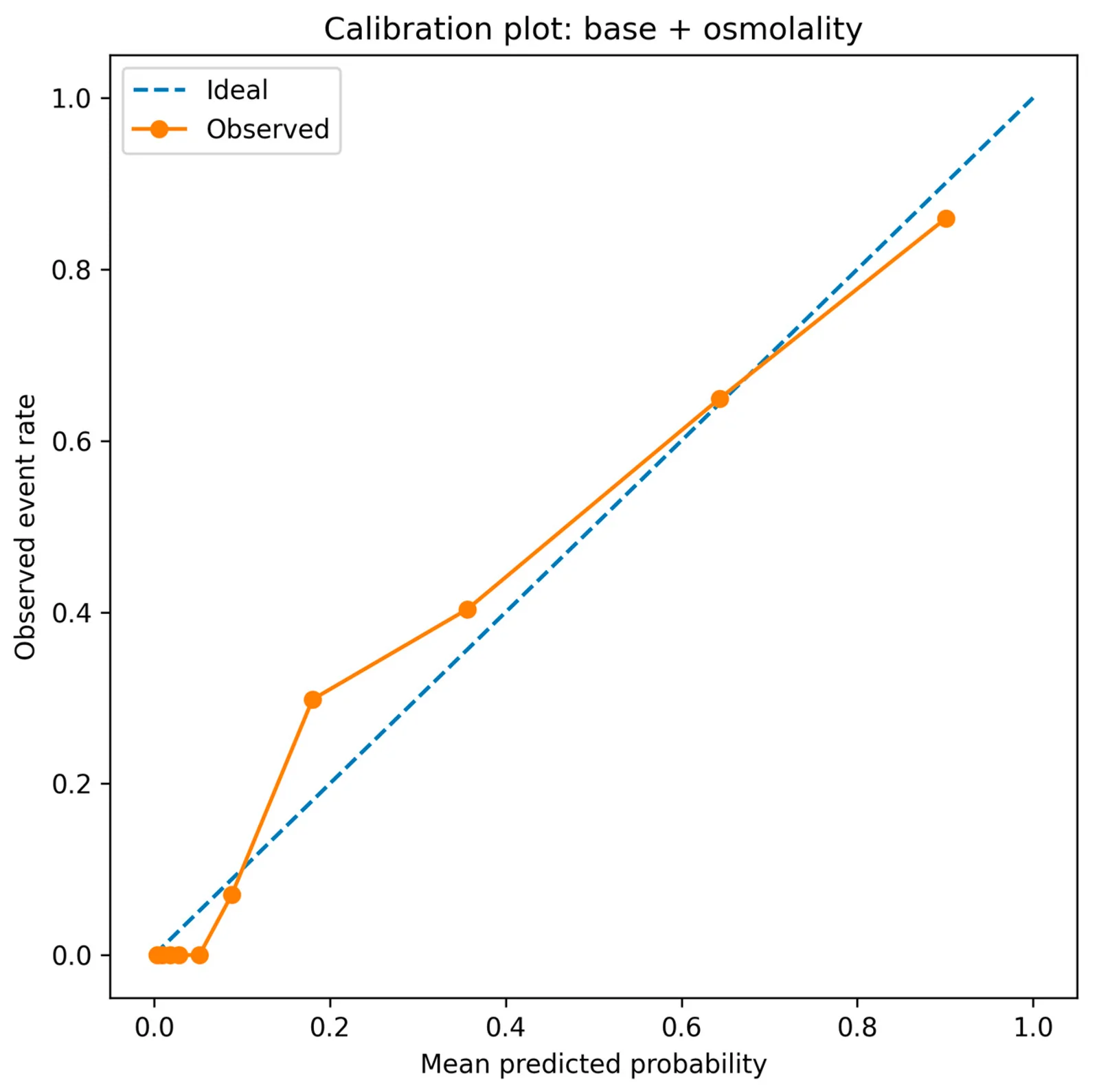
Calibration plot for the base clinical model plus calculated serum osmolality.

The apparent AUC of the base admission model without calculated serum osmolality was 0.927 (95% CI 0.906–0.948). After adding calculated serum osmolality, the apparent AUC was 0.933 (95% CI 0.914–0.952) (Figure 2). Bootstrap internal validation with 1000 resamples yielded an optimism-corrected AUC of 0.924 for the model including osmolality. Apparent calibration assessment showed a calibration slope of 1.00, calibration intercept of 0.00, Brier score of 0.091, and Hosmer–Lemeshow χ^2^ = 13.81 (df = 8; *p* = 0.087) (Table 3; Figure 3).

To evaluate incremental value, the clinically defined base model without calculated serum osmolality was compared with the same model after adding calculated serum osmolality. The increase in AUC was small (ΔAUC = 0.006) and did not reach statistical significance by the DeLong test (*p* = 0.081), whereas the likelihood-ratio test comparing nested models was significant (χ^2^ = 6.28, df = 1; *p* = 0.012). Additional sensitivity analyses compared the composite osmolality model with a model including sodium, glucose, and BUN as separate components and with an expanded clinical model adding diabetes, hypertension, and smoking.

When sodium, glucose, and BUN were entered as separate variables instead of the composite calculated serum osmolality measure, the component model showed an AUC of 0.941. In that model, sodium (OR 1.100 per mmol/L, 95% CI 1.018–1.189; *p* = 0.016), glucose (OR 1.068 per 10 mg/dL, 95% CI 1.028–1.108; *p* < 0.001), and BUN (OR 0.510 per 10 mg/dL, 95% CI 0.317–0.821; *p* = 0.006) were associated with new-onset AF. In an expanded sensitivity model additionally including diabetes, hypertension, and current smoking, the osmolality estimate remained similar (adjusted OR 1.594 per 10 mOsm/kg, 95% CI 1.114–2.280; *p* = 0.011; AUC = 0.934). These findings indicate that calculated serum osmolality summarizes information partly driven by its components and should be interpreted as an integrative admission marker rather than a causal factor.

Calculated serum osmolality alone showed modest discrimination (AUC = 0.655, 95% CI 0.606–0.705). The base clinical model yielded an AUC of 0.927 (95% CI 0.906–0.948), and the model after adding calculated serum osmolality yielded an AUC of 0.933 (95% CI 0.914–0.952).

Apparent calibration assessment for the model including calculated serum osmolality showed a calibration slope of 1.00, a calibration intercept of 0.00, a Brier score of 0.091, and Hosmer–Lemeshow χ^2^ = 13.81 (df = 8; *p* = 0.087).

Complete-case analysis for the primary admission model included 570 patients with 130 new-onset AF events. Variables were selected according to clinical relevance and prior literature rather than univariable *p*-value screening alone. Sodium, glucose, and BUN were not entered individually into the same model containing calculated serum osmolality because they are direct components of the formula. Sensitivity analyses compared the composite osmolality model with models using individual components and alternative renal-function specifications.

Model diagnostics included correlation assessment, VIF values, calibration plot, calibration intercept and slope, Brier score, Hosmer–Lemeshow test, bootstrap optimism-corrected AUC, and incremental comparison of the base model versus the base model plus calculated serum osmolality.

## 4. Discussion

The present study demonstrated that admission calculated serum osmolality was associated with the development of new-onset AF in patients with NSTEMI undergoing PCI. This association should be interpreted in the context of the retrospective design and the requirement for careful temporal sequencing between blood sampling and AF onset. After adjustment for clinically relevant admission covariates, calculated serum osmolality remained associated with new-onset AF per 10 mOsm/kg increase (adjusted OR 1.565, 95% CI 1.102–2.223; *p* = 0.012). The small AUC increase after adding osmolality did not reach statistical significance by the DeLong test, although the likelihood-ratio test was significant. These findings suggest that osmolality may reflect an integrated physiological state involving hydration, metabolic stress, inflammation, renal function, and neurohormonal activation during acute myocardial infarction [10,11].

The component sensitivity analysis showed that sodium, glucose, and BUN entered separately also carried prognostic information, and the component model had numerically higher apparent discrimination than the composite osmolality model. Therefore, calculated serum osmolality should be interpreted as a parsimonious integrative marker derived from routinely available admission variables rather than as evidence that the composite has independent biological effects beyond its components. Similarly, the attenuation of the osmolality estimate in the model without renal-function variables reinforces the need for cautious interpretation and external validation.

The direction of the eGFR association reversed from univariable to multivariable analysis (unadjusted OR 0.676 vs. adjusted OR 1.260 per 10 mL/min/1.73 m^2^). This pattern most likely reflects negative confounding (a suppression effect) rather than a true protective effect of reduced renal function. eGFR equations incorporate age and sex, both of which were strongly associated with new-onset AF and were included in the same model; after adjustment for these variables, the remaining variation in eGFR no longer represents crude renal function. In addition, eGFR is correlated with BUN, which contributes to calculated serum osmolality, so the adjusted eGFR estimate reflects renal function conditional on the osmotic contribution of BUN. The same mechanism likely explains the inverse association of BUN in the component model (OR 0.510 per 10 mg/dL), in which BUN was evaluated alongside eGFR despite higher crude BUN concentrations in patients who developed AF. Consistent with this interpretation, replacing eGFR with serum creatinine yielded an essentially unchanged osmolality estimate (adjusted OR 1.581 per 10 mOsm/kg), and the VIF for eGFR (2.13) did not indicate substantial multicollinearity. Accordingly, the adjusted eGFR and BUN coefficients should not be interpreted causally; they were retained to provide transparent adjustment for renal function.

New-onset AF is one of the most frequent arrhythmic complications of acute myocardial infarction and has been associated with heart failure, thromboembolic events, prolonged hospitalization, and mortality [1,2,3,4]. The mechanisms responsible for AF after myocardial infarction are complex and include ischemia, atrial pressure overload, autonomic nervous system activation, inflammation, oxidative stress, electrolyte abnormalities, and atrial structural remodeling [3,4,5,6,7,19,20,21,22,23]. Rather than representing a single pathway, AF likely reflects the cumulative effects of multiple interacting mechanisms.

Previous studies have reported that increased serum/plasma osmolality is associated with adverse outcomes in ACS, myocardial infarction, heart failure, and critical illness [10,11,13,14]. Rohla et al. showed that plasma osmolality was associated with clinical outcomes in patients with ACS undergoing PCI, and more recent studies have extended the prognostic relevance of osmolality to myocardial infarction and critically ill populations [10,11]. The present study extends this literature by focusing on new-onset AF during NSTEMI hospitalization while using more cautious terminology because association does not itself establish clinical prediction or causality.

Hyperosmolality should not be considered solely a marker of dehydration. Experimental and translational studies indicate that osmotic stress may activate intracellular inflammatory signaling, enhance reactive oxygen species generation, promote endothelial dysfunction, and amplify neurohormonal activation. Animal models of AF have shown that modulation of inflammatory pathways such as NF-κB/HIF-1alpha and NF-κB/MAPK can attenuate atrial remodeling and reduce AF susceptibility [15,16,17,18]. These findings provide biological plausibility for the observed association between admission calculated serum osmolality and new-onset AF.

Inflammation is another central mechanism linking osmolality disturbances with atrial arrhythmogenesis. Patients who developed AF in our cohort had higher hs-CRP concentrations, and inflammatory activation is known to contribute to atrial electrical and structural remodeling through cytokine-mediated alterations in calcium handling, connexin expression, and extracellular matrix turnover [4,5,6,19,20,21,22,23,28]. Pro-inflammatory cytokines, including interleukin-1beta, interleukin-6, tumor necrosis factor-alpha, and transforming growth factor-beta, may promote fibroblast activation and atrial fibrosis, creating structural heterogeneity that facilitates re-entry circuits.

Oxidative stress may also contribute to the observed association. Uric acid was associated with AF in the present analysis and may act as a surrogate marker of oxidative metabolism and xanthine oxidase activity. Increased oxidative stress can impair nitric oxide bioavailability, promote mitochondrial dysfunction, and enhance inflammatory and profibrotic signaling in the atrial myocardium [22,23,29,30,31]. Therefore, concurrent elevations in calculated serum osmolality, hs-CRP, and uric acid may reflect complementary pathophysiological domains rather than isolated risk factors.

Our findings are consistent with previous work from our group highlighting the importance of simple clinical and laboratory markers in AF risk assessment. Bas et al. reported that oxidative stress and inflammatory activation were associated with new-onset AF after ST-segment elevation myocardial infarction (STEMI) [4], Aksoy et al. showed that the C-reactive protein/albumin ratio was associated with new-onset AF after coronary artery bypass grafting (CABG) [5], Bağcı and Aksoy showed that the systemic immune-inflammation index was associated with AF after STEMI [7], Aksoy et al. reported that the CHA2DS2-VASc score was associated with AF after STEMI [8], and Uysal et al. validated the ATRIA and CHA2DS2-VASc scores for predicting AF after CABG [9]. The present study adds calculated serum osmolality as another routinely available marker that may capture metabolic and inflammatory stress during acute myocardial infarction.

From a clinical perspective, calculated serum osmolality is attractive because it can be obtained from routine admission laboratory parameters without additional cost. However, claims regarding improved clinical decision-making or incremental predictive value require formal model comparison and validation. Therefore, we compared a clinically defined base model without osmolality with the same model after adding calculated serum osmolality and reported bootstrap-corrected discrimination and calibration metrics. Because ΔAUC was small and not statistically significant by DeLong testing, the incremental clinical value of osmolality should be considered modest. Notably, the high discrimination of the model was driven largely by clinical covariates (base model AUC 0.927), whereas calculated serum osmolality alone showed only modest discrimination (AUC 0.655); osmolality should therefore not be regarded as a stand-alone predictor of new-onset AF. Until external validation is available, the findings should be considered hypothesis-generating.

The strengths of this study include the evaluation of a low-cost, routinely available biomarker and an analytical approach addressing temporal precedence, clinically justified covariate selection, collinearity diagnostics, sensitivity analyses, internal validation, and calibration. These features support the interpretability of the association between calculated serum osmolality and new-onset AF.

Several limitations should be acknowledged. First, the study was retrospective and single-center, which may limit generalizability. Second, AF was identified during hospitalization; asymptomatic or post-discharge episodes may have been missed, and because in-hospital monitoring was somewhat longer in patients who developed AF, differential detection cannot be fully excluded. Third, calculated serum osmolality was estimated rather than directly measured, and serial osmolality measurements were unavailable. Fourth, echocardiographic variables obtained within 24 h may not uniformly precede AF onset; therefore, admission-only and extended models should be interpreted separately. Fifth, the available de-identified dataset did not include fully reconstructable screening-log counts before final dataset construction; therefore, selection bias cannot be completely excluded. Sixth, the osmolality association was attenuated in the sensitivity model without renal-function variables (*p* = 0.060), suggesting that renal handling of osmotic solutes partly contributes to the observed association. Seventh, 86 patients (13.1%) with missing covariate data were excluded from the complete-case analysis, and missingness was more frequent in patients without AF; although odds ratios from logistic regression are generally robust when missingness depends mainly on the outcome, absolute risk estimates and calibration may have been affected, and bias cannot be fully excluded if missingness was also related to covariate values. Finally, residual confounding by disease severity, renal function, hemodynamic instability, and ischemic burden cannot be excluded despite multivariable adjustment and sensitivity analyses.

## 5. Conclusions

In conclusion, admission calculated serum osmolality was associated with new-onset AF in patients with NSTEMI undergoing PCI. This association may reflect the combined influence of hydration status, metabolic stress, inflammation, oxidative stress, renal function, and hemodynamic vulnerability during acute myocardial infarction. Because calculated serum osmolality is inexpensive and routinely available, it may be useful for further investigation as an adjunctive marker in AF risk assessment. Prospective multicenter studies with external validation are warranted before using osmolality-guided strategies in clinical practice.

## Figures and Tables

**Table 1:** Baseline demographic, clinical, laboratory, and echocardiographic characteristics according to new-onset atrial fibrillation.

Variable	AF (−) (*n* = 520)	AF (+) (*n* = 136)	*p* Value
Clinical characteristics			
Age, years	61.1 ± 12.7	68.2 ± 11.7	<0.001
Female sex, *n* (%)	80 (15.4)	58 (42.6)	<0.001
Diabetes mellitus, *n* (%)	122 (23.5)	48 (35.3)	0.007
Hypertension, *n* (%)	202 (38.8)	98 (72.1)	<0.001
Hyperlipidemia, *n* (%)	118 (22.7)	32 (23.5)	0.926
Current smoker, *n* (%)	342 (65.8)	60 (44.1)	<0.001
Obesity, *n* (%)	178 (34.2)	64 (47.1)	0.008
Heart rate, bpm	76.8 ± 17.1	77.6 ± 22.7	0.708
Systolic blood pressure, mmHg	124.0 ± 26.9	109.2 ± 31.0	<0.001
Diastolic blood pressure, mmHg	74.2 ± 14.6	64.8 ± 19.3	<0.001
Weight, kg	74.2 ± 12.0	78.9 ± 18.7	0.007
Height, cm	167.2 ± 6.9	161.9 ± 9.2	<0.001
Body mass index, kg/m^2^	26.1 ± 3.6	29.4 ± 5.2	<0.001
Waist circumference, cm	91.7 ± 7.4	98.1 ± 9.9	<0.001
Laboratory findings			
Peak CK-MB, U/L	152.5 (86.0–278.0)	231.5 (97.2–340.0)	<0.001
Peak Troponin, ng/mL	3.6 (1.5–6.9)	3.8 (2.7–8.3)	0.002
Hemoglobin, g/dL	14.6 ± 2.1	13.1 ± 2.7	<0.001
Glucose, mg/dL	163.9 ± 84.9	224.0 ± 112.1	<0.001
Creatinine, mg/dL	1.1 ± 0.3	1.2 ± 0.3	<0.001
Blood urea nitrogen, mg/dL	18.8 ± 7.4	22.4 ± 6.1	<0.001
Total cholesterol, mg/dL	178.9 ± 42.3	165.2 ± 43.1	0.001
Triglycerides, mg/dL	120.0 (87.0–172.0)	122.0 (87.0–141.5)	0.189
HDL cholesterol, mg/dL	41.0 ± 9.2	40.3 ± 7.8	0.368
LDL cholesterol, mg/dL	109.3 ± 35.6	100.4 ± 35.9	0.011
High-sensitivity C-reactive protein, mg/L	12.0 (6.2–29.8)	73.0 (30.2–100.0)	<0.001
Gamma-glutamyl transferase, U/L	25.0 (18.0–36.8)	42.0 (26.0–52.0)	<0.001
Uric acid, mg/dL	6.0 ± 1.4	7.9 ± 1.2	<0.001
Sodium, mmol/L	137.9 ± 7.4	138.5 ± 3.8	0.217
Potassium, mmol/L	4.4 ± 0.5	4.7 ± 0.6	<0.001
Calculated serum osmolality, mOsm/kg	291.6 ± 15.7	297.4 ± 8.9	<0.001
Echocardiographic findings			
Left ventricular ejection fraction, %	45.1 ± 9.5	38.0 ± 10.3	<0.001
Left atrial diameter, mm	39.4 ± 3.4	42.3 ± 2.7	<0.001

Values are presented as mean ± standard deviation, median (interquartile range), or number (percentage), according to variable distribution. Normally distributed continuous variables were compared using the independent-samples Student’s *t*-test, non-normally distributed variables using the Mann–Whitney U test, and categorical variables using the chi-square test or Fisher’s exact test. Abbreviations: AF, atrial fibrillation; CK-MB, creatine kinase-MB; HDL, high-density lipoprotein; LDL, low-density lipoprotein.

**Table 2:** Clinically prespecified univariable and multivariable admission logistic regression analyses for new-onset atrial fibrillation.

Variable	Univariable OR (95% CI)	*p* Value	Multivariable OR (95% CI)	*p* Value	VIF
Age, per year	1.051 (1.033–1.069)	<0.001	1.039 (1.008–1.070)	0.013	1.81
Female sex	4.258 (2.770–6.545)	<0.001	4.050 (1.967–8.339)	<0.001	1.39
BMI, per kg/m^2^	1.205 (1.146–1.267)	<0.001	1.187 (1.091–1.292)	<0.001	1.36
SBP, per mmHg	0.982 (0.975–0.989)	<0.001	0.973 (0.962–0.984)	<0.001	1.19
Heart rate, per bpm	1.003 (0.993–1.013)	0.579	1.030 (1.015–1.046)	<0.001	1.10
eGFR, per 10 mL/min/1.73 m^2^	0.676 (0.610–0.749)	<0.001	1.260 (1.035–1.532)	0.021	2.13
Potassium, per mmol/L	3.006 (2.092–4.319)	<0.001	1.300 (0.756–2.236)	0.342	1.22
hs-CRP, per 10 mg/L	1.402 (1.316–1.493)	<0.001	1.207 (1.104–1.320)	<0.001	1.37
Uric acid, per mg/dL	2.825 (2.321–3.439)	<0.001	2.205 (1.717–2.831)	<0.001	1.58
Calculated serum osmolality, per 10 mOsm/kg	1.923 (1.536–2.407)	<0.001	1.565 (1.102–2.223)	0.012	1.12

**Table 3:** Model performance, internal validation, calibration, and incremental value of calculated serum osmolality.

Metric	Result
Primary complete-case population	570 patients; 130 new-onset AF events
**Base admission model without osmolality**	AUC 0.927 (95% CI 0.906–0.948); optimism-corrected AUC 0.919; Brier score 0.092; Hosmer–Lemeshow χ^2^ = 11.30 (df = 8), *p* = 0.185; apparent calibration slope/intercept = 1.00/0.00
**Base admission model + calculated serum osmolality**	AUC 0.933 (95% CI 0.914–0.952); optimism-corrected AUC 0.924; Brier score 0.091; Hosmer–Lemeshow χ^2^ = 13.81 (df = 8), *p* = 0.087; apparent calibration slope/intercept = 1.00/0.00
**Incremental value of osmolality**	ΔAUC = 0.006; DeLong *p* = 0.081; likelihood-ratio χ^2^ = 6.28, df = 1, *p* = 0.012
**Main osmolality estimate**	Adjusted OR 1.565 per 10 mOsm/kg (95% CI 1.102–2.223), *p* = 0.012; VIF = 1.12
**Creatinine sensitivity model**	Adjusted OR for osmolality 1.581 per 10 mOsm/kg (95% CI 1.112–2.248), *p* = 0.011; AUC = 0.934
**No renal-function sensitivity model**	Adjusted OR for osmolality 1.364 per 10 mOsm/kg (95% CI 0.986–1.885), *p* = 0.060; AUC = 0.931
**Extended echocardiographic sensitivity model**	*n* = 560; AUC = 0.948; adjusted OR for osmolality 1.543 per 10 mOsm/kg (95% CI 1.073–2.218), *p* = 0.019. This was not used as the primary model because echocardiographic timing may not have preceded AF onset in all patients.
Component model with sodium, glucose, and BUN entered separately	AUC 0.941; sodium OR 1.100 per mmol/L (95% CI 1.018–1.189), *p* = 0.016; glucose OR 1.068 per 10 mg/dL (95% CI 1.028–1.108), *p* < 0.001; BUN OR 0.510 per 10 mg/dL (95% CI 0.317–0.821), *p* = 0.006; likelihood-ratio χ^2^ vs. base model = 21.54, df = 3, *p* < 0.001.
Expanded clinical sensitivity model adding diabetes, hypertension, and smoking	Adjusted OR for osmolality 1.594 per 10 mOsm/kg (95% CI 1.114–2.280), *p* = 0.011; AUC = 0.934. Hypertension remained associated with AF, whereas diabetes and smoking were not significant after adjustment.
**Interpretation**	Calculated serum osmolality remained associated with new-onset AF in the primary model. The AUC increase was small and not significant by DeLong testing; therefore, its incremental clinical value should be considered modest.

## Data Availability

The data that support the findings of this study are not publicly available due to privacy and ethical restrictions. De-identified data may be made available by the corresponding author upon reasonable request and with permission from the Institutional Ethics Committee, where applicable.

## Abbreviations

The following abbreviations are used in this manuscript:

Abbreviations	Definition
ACS	Acute coronary syndrome
AF	Atrial fibrillation
AUC	Area under the curve
BMI	Body mass index
BUN	Blood urea nitrogen
CABG	Coronary artery bypass grafting
CI	Confidence interval
CK-MB	Creatine kinase-MB
CRP	C-reactive protein
ECG	Electrocardiography
eGFR	Estimated glomerular filtration rate
ESC	European Society of Cardiology
GGT	Gamma-glutamyl transferase
HDL	High-density lipoprotein
hs-CRP	High-sensitivity C-reactive protein
IQR	Interquartile range
LA	Left atrium
LDL	Low-density lipoprotein
LVEF	Left ventricular ejection fraction
MAPK	Mitogen-activated protein kinase
NF-κB	Nuclear factor kappa B
NSTEMI	Non-ST-segment elevation myocardial infarction
OR	Odds ratio
PCI	Percutaneous coronary intervention
ROC	Receiver operating characteristic
SBP	Systolic blood pressure
SD	Standard deviation
VIF	Variance inflation factor
